# Out-of-hospital cardiac arrest detection by a wearable: the first real-life case

**DOI:** 10.1016/j.resplu.2026.101245

**Published:** 2026-01-29

**Authors:** Roos Edgar, Catharina E. Jansen, Lente R. Pol, Ron Pisters, Niels van Royen, Judith L. Bonnes

**Affiliations:** a Department of Cardiology, Radboud University Medical Center, Geert Grooteplein Zuid 10, 6525 GA Nijmegen, the Netherlands; b Department of Cardiology, Rijnstate Hospital, Wagnerlaan 5, 6815 AD Arnhem, the Netherlands

**Keywords:** Out-of-hospital cardiac arrest, Automated detection, Wearable, Photoplethysmography, Accelerometry

## Abstract

**Introduction:**

Automated cardiac arrest detection and alerting using wearable technology is an emerging innovation in resuscitation science. The DETECT program aims to develop a wristband that integrates photoplethysmography and accelerometry to identify out-of-hospital cardiac arrest events and alert the emergency medical services. We report the first case in which the algorithm successfully detected a cardiac arrest that occurred during daily life in an implantable cardioverter-defibrillator (ICD) patient.

**Case description:**

A 64-year old male with a secondary prevention ICD participated in the DETECT-3 study. As part of the study, he was wearing the CardioWatch wristband during daily life for a two-month period. While cycling, the patient experienced a ventricular fibrillation cardiac arrest that was successfully terminated by the ICD. The cardiac arrest detection algorithm of the CardioWatch correctly recognized the cardiac arrest event and subsequent collapse based on photoplethysmography and accelerometry data during post-processing. The patient was admitted for rhythm observation and made a good recovery.

**Discussion:**

This first real-life case shows feasibility of cardiac arrest detection using wrist-derived photoplethysmography and accelerometry. Further studies are warranted to validate the algorithm in additional cardiac arrest cases, and to minimize false positive alerts during daily-life use. Once implemented, automated cardiac arrest detection and alerting has the potential to shorten treatment delays and improve survival.

## Highlights

• Report of first real-life OHCA detected by wristband-integrated sensors.
• A photoplethysmography-model detected pulselessness within seconds during cycling.
• Accelerometry-based fall detection model confirmed cardiac arrest-related collapse.
• Wearable detection may enable timely EMS activation in unwitnessed OHCA.

## Introduction

Survival from out-of-hospital cardiac arrest depends on immediate recognition and early start of cardiopulmonary resuscitation.^1^, ^2^ Although survival from witnessed cardiac arrest has increased due to widespread automated external defibrillator and citizen responder networks, survival from unwitnessed out-of-hospital cardiac arrest remains dismal (<5%) as help often arrives too late.^1^, ^3^ Automated cardiac arrest detection using wearable technology may help to bridge the survival gap between witnessed and unwitnessed out-of-hospital cardiac arrest.^4^

Several research groups, including DETECT, are developing wearables such as wristbands and rings for automated cardiac arrest detection and alerting.^5^, ^6^, ^7^, ^8^ Within DETECT, a photoplethysmography-based cardiac arrest detection algorithm was previously developed with 98% sensitivity in induced arrests.^9^ It is now being validated and refined in the DETECT-3 study to minimize false positives during daily-life use.

To date, cases of cardiac arrest during daily life while wearing a wristband with automated cardiac arrest detection functionality have not been described in the literature. We report the first case of a DETECT-3 study participant with an implantable cardioverter-defibrillator (ICD) who experienced a ventricular fibrillation cardiac arrest, while wearing our wristband.

## Case description

A 64-year old male with heart failure with reduced ejection fraction and permanent atrial fibrillation, participated in the DETECT-3 study. His medical history reports implantation of a Biotronik Acticor 7 VR-T single chamber ICD 2.5 years prior to study inclusion, following episodes of sustained ventricular tachycardia. The ICD was programmed with a ventricular tachycardia detection zone (182–230 bpm) with two antitachycardia pacing sequences before shock therapy, and a ventricular fibrillation zone (≥231 bpm) with one antitachycardia pacing attempt prior to shock therapy. Before study participation, the patient never received ICD therapy. At the time of inclusion, the patient did not have complains of chest pain, palpitations, or shortness of breath.

As part of the study DETECT-3 protocol, he wore the CardioWatch wristband for a two-month period during daily life. The CE-certified CardioWatch 287-2 (Corsano Health, the Hague, the Netherlands) continuously registers photoplethysmography and accelerometry signals, which are automatically send to a protected cloud-based study portal. Photoplethysmography sensors use light to measure changes in blood volume in the microvasculature, allowing continuous estimation of the heart rate. Accelerometry sensors measure movement of the wrist in three directions. The collected data from the wristband will be analyzed after study completion to assess the false positive cardiac arrest alarm rate, using our earlier developed algorithm.^9^

**Fig. 1:**
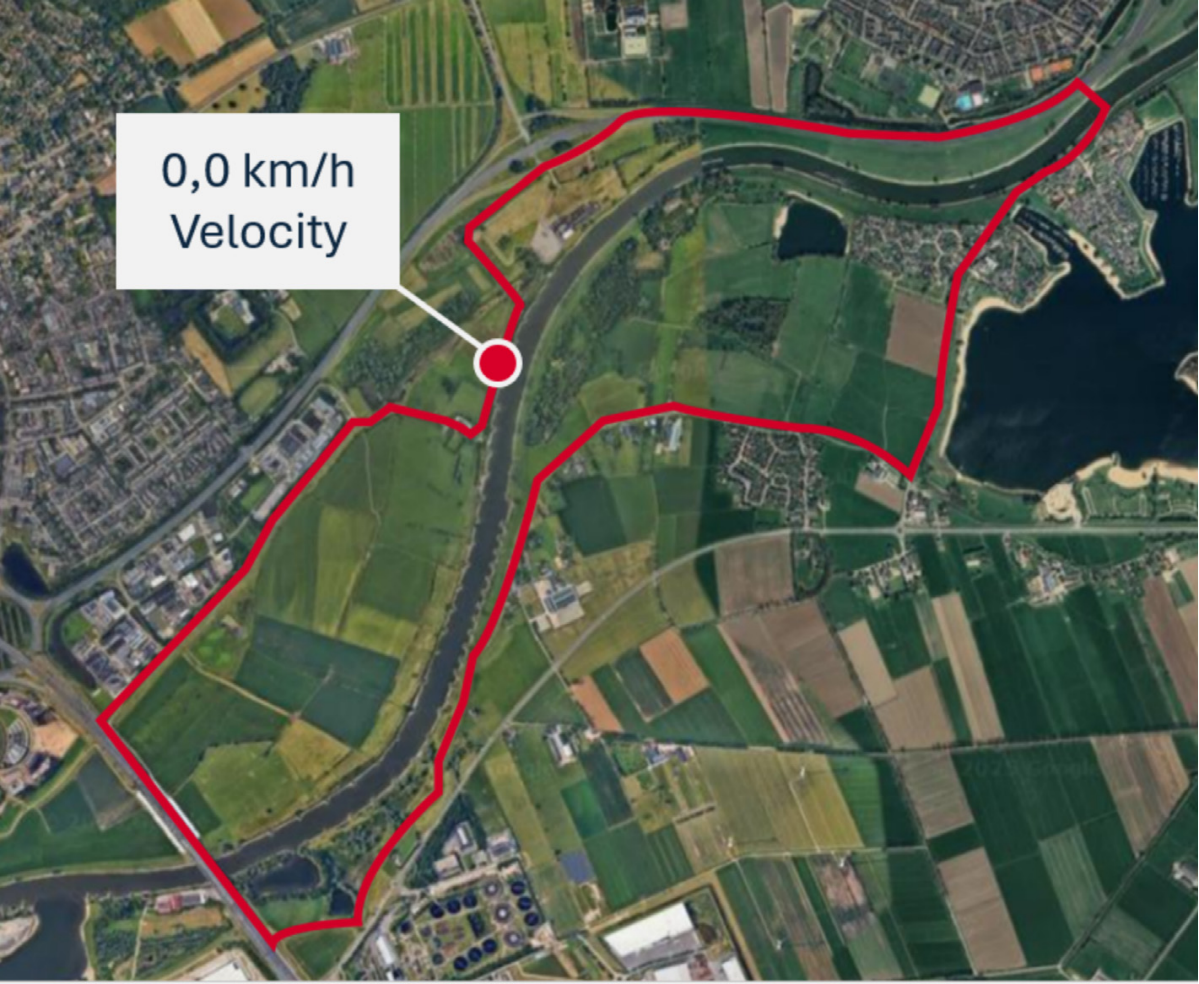
**The cycling route recorded by the patient’s personal smartwatch, illustrating that the cardiac arrest occurred during daily life. The red dot indicates the location and time of collapse, confirmed by a timestamp showing a velocity of 0 km/h**. (For interpretation of the references to color in this figure legend, the reader is referred to the web version of this article.)

**Fig. 2:**
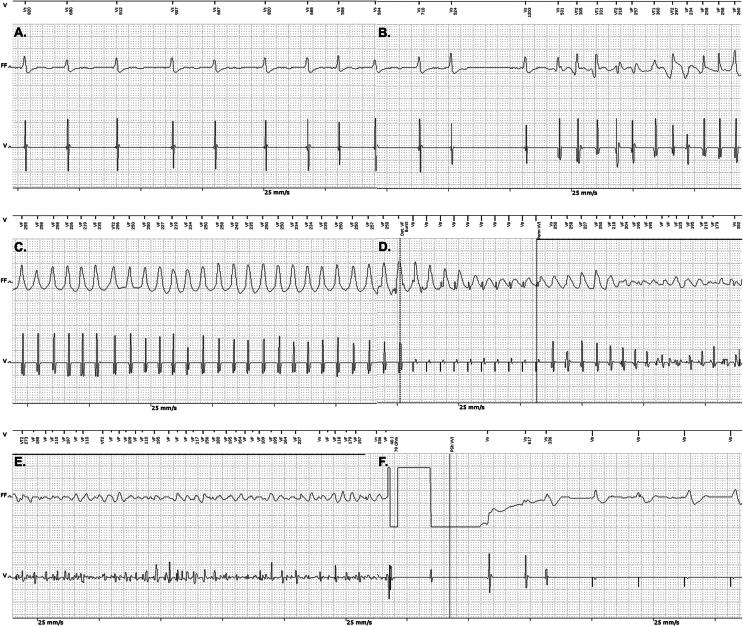
**Electrograms interrogated from the ICD during the arrhythmia, antitachycardia pacing, and shock therapy. Above each electrogram, the detection zone (Vs, VT1, VT2, or VF) and corresponding RR interval are displayed. In each recording, the upper trace on the ECG paper represents the far-field channel (FF) and the lower trace corresponds to the ventricular channel (V; tip-ring). All recordings are shown at a paper speed of 25 mm/s. (A) Atrial fibrillation, (B) conversion from atrial fibrillation to a ventricular tachycardia, (C) monomorphic ventricular tachycardia with a heart rate falling in the ventricular fibrillation zone, (D) antitachycardia pacing burst after which the arrhythmia accelerates to ventricular fibrillation, (E) ventricular fibrillation, (F) successful defibrillatory shock and post-shock pacing afterwards**. ICD = implantable cardioverter-defibrillator,

After three weeks of study participation, the patient received an ICD shock. An arrhythmia occurred while the patient was cycling, causing a fall from his bike and leaving the patient unconscious. After receiving the ICD therapy, the patient regained consciousness and returned home by himself. The family of the patient informed the investigators about the date and estimated time of the ICD shock. During the incident, the patient was wearing another smartwatch that recorded his cycling route, illustrating that the cardiac arrest occurred during daily life. A timestamp showing a velocity of 0 km/h confirmed the location and time of the fall during postprocessing (Fig. 1). The patient was admitted to the hospital where no triggering factors for the arrhythmia could be identified. The ICD was interrogated showing that the patient developed a monomorphic ventricular tachycardia with a heart rate falling in the ventricular fibrillation zone. A single antitachycardia pacing burst was delivered, after which the arrhythmia accelerated into ventricular fibrillation. Following a charge time of nine seconds, a 40 J shock was applied, successfully terminating ventricular fibrillation. Post-shock pacing was subsequently initiated. See Fig. 2.

**Fig. 3:**
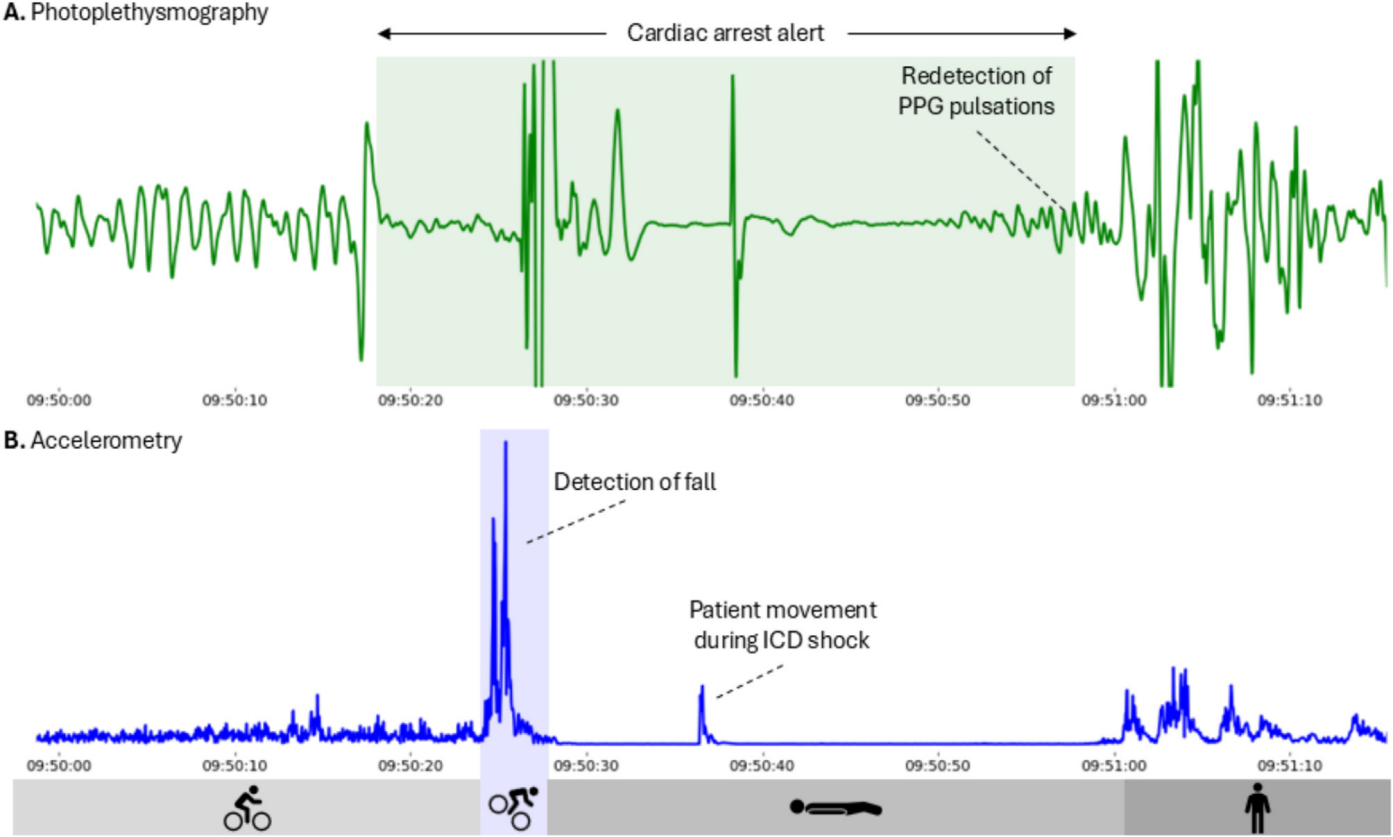
**Photoplethysmography and accelerometry signals during the cardiac arrest event. (A) On the left, normal photoplethysmography pulsations are visible; every pulse corresponds to one heartbeat. After approximately 20 s, there is no pulsatile flow, corresponding to the cardiac arrest event. The green box indicates the period of the cardiac arrest alert generated by the photoplethysmography-algorithm. (B) The accelerometer signal, with every spike corresponding to user movement. A flat line corresponds to no movement. The blue box indicates the fall of the patient, as detected by the fall detection algorithm. Afterwards, the patient lies still for approximately 30 s. After return of pulsations in the photoplethysmography signal, the patient starts moving again**. ICD = implantable cardioverter defibrillator. (For interpretation of the references to color in this figure legend, the reader is referred to the web version of this article.)

The 32 Hz raw photoplethysmography and accelerometry data from the CardioWatch wristband were exported from the study cloud and analyzed for cardiac arrest events. At the expected time of the arrhythmia, an absence of photoplethysmography pulsations is visible, see Fig. 3. The accelerometry signal shows a large acceleration followed by a period of no movement. A data scientist at Corsano Health, who was blinded to the cardiac arrest event, ran the data through the photoplethysmography-based cardiac arrest detection algorithm. In short, the photoplethysmography-based algorithm detects the absence of pulsations based on signal amplitude and specific morphologic features of the photoplethysmography waveform within five seconds. The workflow of the algorithm has been described earlier.^9^ Cardiac arrest was detected at 09:50:17, after a loss of pulse in the photoplethysmography signal. The alert was terminated at 09:50:57, when the return of several normal photoplethysmography peaks was detected. Moreover, the fall with a period of no movement afterwards was detected by our previously developed fall detection algorithm. The fall occurred after approximately 8–10 s of pulselessness. Furthermore, after administration of the ICD shock, a slow return of photoplethysmography pulsations was observed at 10 s, with physical movement at 20 s.

Written informed consent was obtained from the patient for publication of this report.

## Discussion

In this case report, we describe the first successful detection of real-life cardiac arrest using wristband-integrated photoplethysmography and accelerometry sensors, based on retrospective analysis of wearable data. These results are promising for further development of this awaited technology.

The case illustrates the feasibility of photoplethysmography and accelerometry signals for wearable-based cardiac arrest detection during daily-life use. Although photoplethysmography quality is typically disturbed by motion,^10^ adequate photoplethysmography quality is seen in this patient during cycling (see Fig. 3). The loss of photoplethysmography pulsations is clearly visible during cycling as well. Subsequently, there was an 8–10 s delay between the loss of pulsations and the fall. This delay is consistent with literature on the interval between sudden loss of cardiac output and syncope.^11^ The described case demonstrates that the accelerometer sensor has the potential to confirm the likelihood of a cardiac arrest, by detecting both the fall and period of no movement afterwards. After successful termination of ventricular fibrillation, there was a slow return of photoplethysmography pulsations during post-shock pacing.

Patients with an increased risk of sudden cardiac death who are not protected by an ICD represent a potential target group for a wristband with automated cardiac arrest detection. In the presented case, the patient was cycling alone when the arrhythmia began. Without an ICD, the patient’s survival would have depended entirely on the presence and response of bystanders. Early recognition and call for help is the most important link in the chain of survival as the survival chance decreases by 5–10% for every minute of delay.^4^, ^12^, ^13^ A wristband that can autonomously activate the emergency medical chain could act as a first witness. This case illustrates a promising use case for such technology.

Several initiatives are working towards wearable-based cardiac arrest detection.^14^, ^15^ While the currently developed algorithms have shown promise in controlled environments, they lack validation in real-world conditions. One commercially available system reported a 67% sensitivity for cardiac arrest detection, though it was validated only in simulated settings.^8^ This case study is the first to demonstrate that shockable cardiac arrest occurring during daily life can be detected using photoplethysmography and accelerometry. In previous studies, data were collected from patients and healthy volunteers who did not move and with the investigator attaching the wearable to ensure sufficient sensor-skin contact.^6^, ^7^, ^14^ However, in everyday use, photoplethysmography is easily affected by noise, particularly during motion.^10^, ^16^ Therefore, large-scale testing in real-life settings is an important step in the development of this technology.

The multidisciplinary DETECT program aims to develop a comprehensive wearable-based technology for automated cardiac arrest detection and alerting, covering everything from technological development and validation in large patient cohorts to integration with local emergency medical systems. In future studies, we will provide external validation of algorithm performance in both shockable and non-shockable cardiac arrest (sensitivity) and refine the algorithm based on data from daily-life use to reduce false alerts (specificity).^5^ Occurrence of false positive alerts in the described case will be assessed as part of the DETECT-3 main analysis. The DETECT-3 study also involves integration of accelerometry data into the cardiac arrest detection algorithm, with the aim to either confirm the likelihood of a cardiac arrest (as was seen in this case) or to rule out an arrest in case of ongoing movement.

Automated cardiac arrest detection through wearable technology will only impact out-of-hospital cardiac arrest survival if an effective emergency medical response is deployed. Therefore, seamless integration with local emergency systems is part of the project. The connection between the CardioWatch and the Dutch citizen responder network is currently under development. Once established and the algorithm operates on the device, cardiac arrest alerts will autonomously activate the emergency medical system. Finally, the detection model will be tested in ICD patients, including the alerting function.

## Conclusion

This report presents the first real-life out-of-hospital cardiac arrest case that was successfully detected using wristband-based photoplethysmography and accelerometry. The findings highlight the promise of this potentially lifesaving technology. Future research should focus on validating accuracy in daily life and ensuring seamless integration with emergency medical services. When implemented, automated cardiac arrest detection and alerting has the potential to shorten treatment delays, enabling timely help for both witnessed and unwitnessed cases.

## CRediT authorship contribution statement

**Roos Edgar:** Writing – original draft, Visualization, Methodology, Investigation, Data curation, Conceptualization. **Catharina E. Jansen:** Writing – review & editing, Investigation, Data curation. **Lente R. Pol:** Writing – original draft, Visualization, Investigation, Data curation. **Ron Pisters:** Writing – review & editing, Investigation. **Niels van Royen:** Writing – review & editing, Supervision, Resources. **Judith L. Bonnes:** Writing – review & editing, Validation, Supervision, Resources, Methodology, Conceptualization.

## Ethics approval and consent to participate

The DETECT-3 study protocol was reviewed by the Medical Ethics Review Committee Netherlands East, which issued a non-WMO declaration. The patient provided written informed consent for study participation. Additionally, written informed consent was obtained from the patient for publication of this case report.

## Funding

DETECT-3 is funded by public–private partnerships allowance (grant number R0007420) made available by Top Sector Life Science & Health to Radboudumc.

## Declaration of competing interest

The authors declare the following financial interests/personal relationships which may be considered as potential competing interests: Niels van Royen reports financial support was provided by Radboud University Medical Center. Niels van Royen reports a relationship with Biotronik, Abbott, Medtronic, Philips that includes: funding grants. Niels van Royen reports a relationship with Bayer, RainMed, Microport that includes: speaking and lecture fees. Roos Edgar is Young Investigator Editorial Board member of Resuscitation Plus. If there are other authors, they declare that they have no known competing financial interests or personal relationships that could have appeared to influence the work reported in this paper.

## Data Availability

Data sharing is possible after publication of the full study. Data will be made available on the Radboud Data Repository. The data will be shared with investigators whose proposed use of the data has been approved by the review committee identified for this purpose.
